# Supplementary material for: Estimating orientation in natural scenes: A spiking neural network model of the insect central complex
Source: PLoS Comput Biol. 2024 Aug 15;20(8):e1011913. doi: 10.1371/journal.pcbi.1011913 (PMC11349202; doi:10.1371/journal.pcbi.1011913)
Supplement: S1 Fig — (A) 14 R2 and 7 R4 averaged Drosophila receptive fields [28]. (B) 26 synthetic R4 receptive fields with hexagonal spacing. (C) 26 synthetic R4 receptive fields with square grid spacing. (D) 26 synthetic R4 receptive fields random symmetrical spacing. Each panel is applied as a filter to 95 x 360 degree panoramas. (PDF) [file pcbi.1011913.s001.pdf]

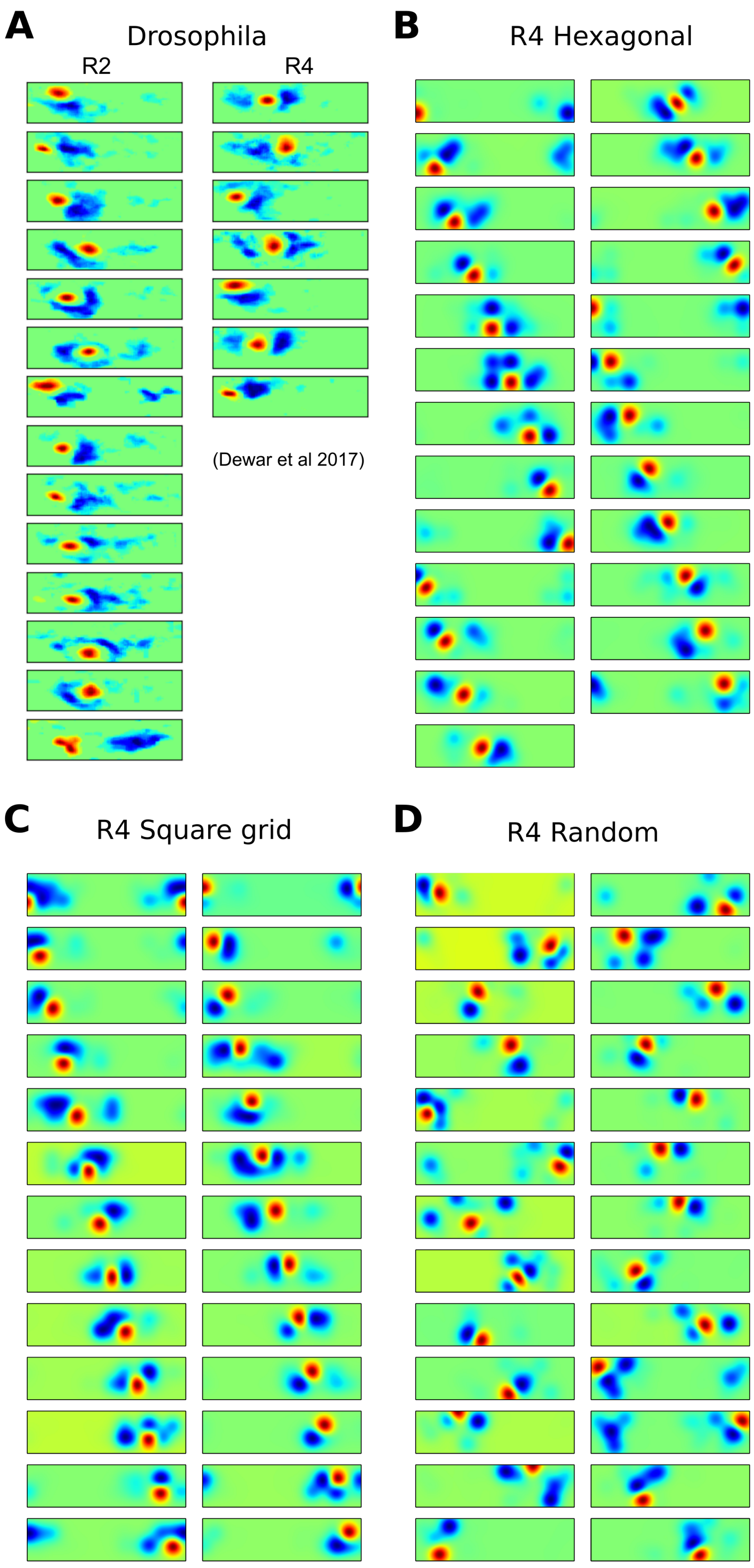

Fig S1: **Ring neuron receptive fields.** (A) 14 R2 and 7 R4 averaged Drosophila receptive fields [9]. (B) 26 synthetic R4 receptive fields with hexagonal spacing. (C) 26 synthetic R4 receptive fields with square grid spacing. (D) 26 synthetic R4 receptive fields random symmetrical spacing. Each panel is applied as a filter to 95 x 360 degree panoramas.
