# Supplementary material for: Estimating orientation in natural scenes: A spiking neural network model of the insect central complex
Source: PLoS Comput Biol. 2024 Aug 15;20(8):e1011913. doi: 10.1371/journal.pcbi.1011913 (PMC11349202; doi:10.1371/journal.pcbi.1011913)
Supplement: S2 Fig — One example frame from each of the 33 panoramic videos of natural scenes. Videos were captured at various locations across the University of Sussex and adjacent Stanmer Park. (top) 23 rotation only videos. For 3 locations examples were captured in open areas on both sunny and overcast days (total 6 panoramas). The remaining examples include trees either in a woodland or campus setting which occlude some or all of the sky. (bottom) 10 circling videos. A photograph of the Spidercam showing 4 cables connected to the camera assembly. Cable lengths are changed by winding or unwinding the cables from a spool using stepper motors, in order to move the camera in 3 dimensions. For all panoramas the performance group (high variance, low variance, or high failure), and the approximate distance to closest landmark is indicated on the y-axis. (PDF) [file pcbi.1011913.s002.pdf]

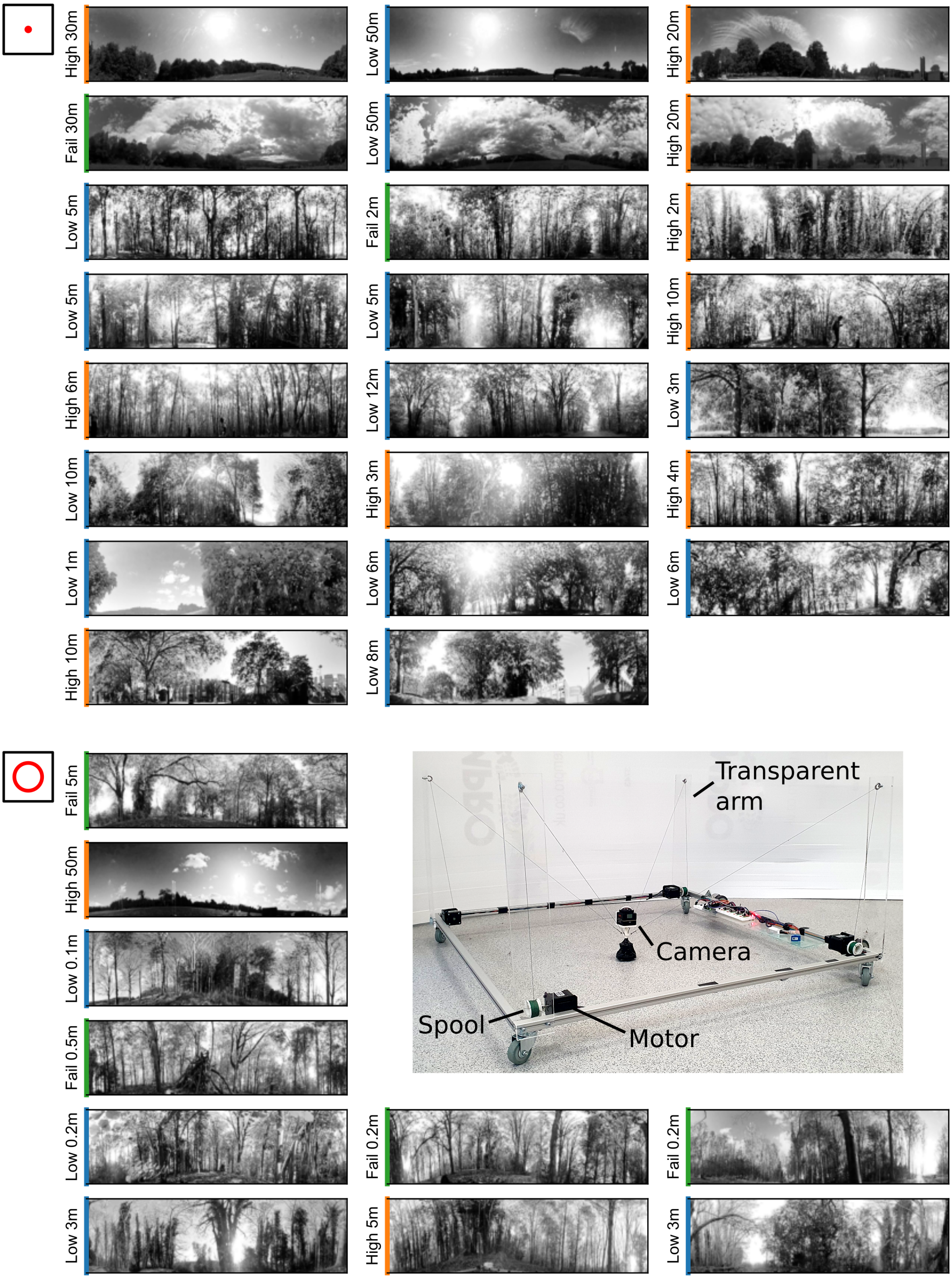

Fig S2: **Natural scenes.** One example frame from each of the 33 panoramic videos of natural scenes. Videos were captured at various locations across the University of Sussex and adjacent Stanmer Park. (top) 23 rotation only videos. For 3 locations examples were captured in open areas on both sunny and overcast days (total 6 panoramas). The remaining examples include trees either in a woodland or campus setting which occlude some or all of the sky. (bottom) 10 circling videos. A photograph of the Spidercam showing 4 cables connected to the camera assembly. Cable lengths are changed by winding or unwinding the cables from a spool using stepper motors, in order to move the camera in 3 dimensions. For all panoramas the performance group (high variance, low variance, or high failure), and the approximate distance to closest landmark is indicated on the y-axis.
