# Supplementary material for: Estimating orientation in natural scenes: A spiking neural network model of the insect central complex
Source: PLoS Comput Biol. 2024 Aug 15;20(8):e1011913. doi: 10.1371/journal.pcbi.1011913 (PMC11349202; doi:10.1371/journal.pcbi.1011913)
Supplement: S3 Fig — (A) Logged mean error for the final rotation for all simulations across rotation only natural scenes, for combinations of parameters learning rate (η) and ρ (see Methods for learning rule). Parameters η = 0.01 and ρ = 0.06 were selected from the dark blue region of low error (white star). Colourbar indicates logged mean error over all natural scenes in degrees. (B) Average error across all natural scenes when learning remains on or turned off during the probe trial (T-test p = 0.001). (C) Percentage of weights changed over multiple revolutions of learning for Angular Velocity = 60 deg/s, η = 0.01 and ρ = 0.06. (D) Developments in the weight space over 10 revolutions. After 5 revolutions the weight space is fully developed and very few changes occur. (PDF) [file pcbi.1011913.s003.pdf]

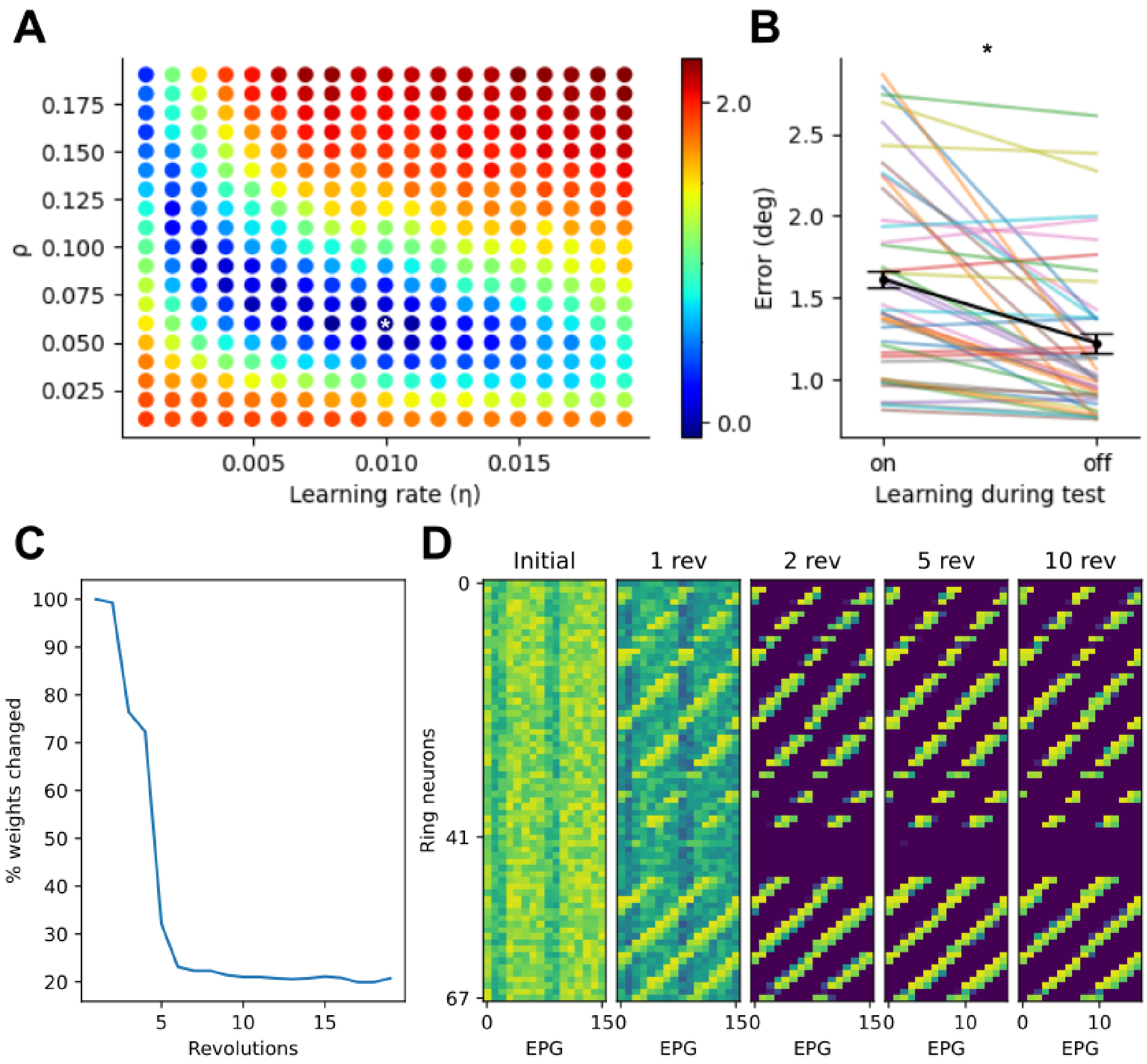

Fig S3: **Varying the Learning parameters** (A) Logged mean error for the final rotation for all simulations across rotation only natural scenes, for combinations of parameters learning rate (η) and ρ (see Methods for learning rule). Parameters $\eta = 0.01$ and $\rho = 0.06$ were selected from the dark blue region of low error (white star). Colourbar indicates logged mean error over all natural scenes in degrees. (B) Average error across all natural scenes when learning remains on or turned off during the probe trial (T-test p = 0.001). (C) Percentage of weights changed over multiple revolutions of learning for Angular Velocity = 60 deg/s, $\eta = 0.01$ and $\rho = 0.06$. (D) Developments in the weight space over 10 revolutions. After 5 revolutions the weight space is fully developed and very few changes occur.
