# Supplementary material for: Estimating orientation in natural scenes: A spiking neural network model of the insect central complex
Source: PLoS Comput Biol. 2024 Aug 15;20(8):e1011913. doi: 10.1371/journal.pcbi.1011913 (PMC11349202; doi:10.1371/journal.pcbi.1011913)
Supplement: S4 Fig — (A) In each row and column, the number of bump rotations for a single run of the model for a specific combination of weights is represented by colour (darker = more rotations; empty means weight combination was not viable). Bump rotations are measured by unwrapping the head direction estimate and finding the total degrees turned. On the x-axis, there are 7 sets of 7 columns. Each set of 7 columns has a fixed weight from R to EPG cell ranging from -0.5 to -5, shown on top x-axis. Within each set, the weight from Δ7 cells to EPG cells cycles through the same 7 values between -0.5 and -5, shown on the bottom x-axis. On the y axis, the weight from EPG to PEN cells varies every 10 sets of 10 rows (range 0.05 to 0.23 in steps of 0.02; shown on left y-axis) while the weight from PEN to EPG cells changes every cell (cycling in 10 steps from 0.05 to 0.23), shown on the right y-axis. Only parameters that meet the following conditions were considered viable: the bump must persist for the full simulation; the bump must be at least 2 cells wide and no wider than 8 cells; and the bump must move at least one full rotation within a 20000ms simulation. The parameter set in Table 1 results in 3 rotations in 20000ms. Selecting alternative parameters could allow different ranges of angular velocity to be accurately tracked. For all parameter combinations the weights from EPG to R, EPG to Δ7 and EPG to EPG are set to constant values specified in Table 1. (B) Number of rotations produced by the model when varying EPG to R weights (between 0.005 to 0.05 in steps of 0.005; x-axis). In each subpanel, the weight between two other groups of cells is varied (y-axis). The weights that were not varied were fixed to the values shown in Table 1. (PDF) [file pcbi.1011913.s004.pdf]

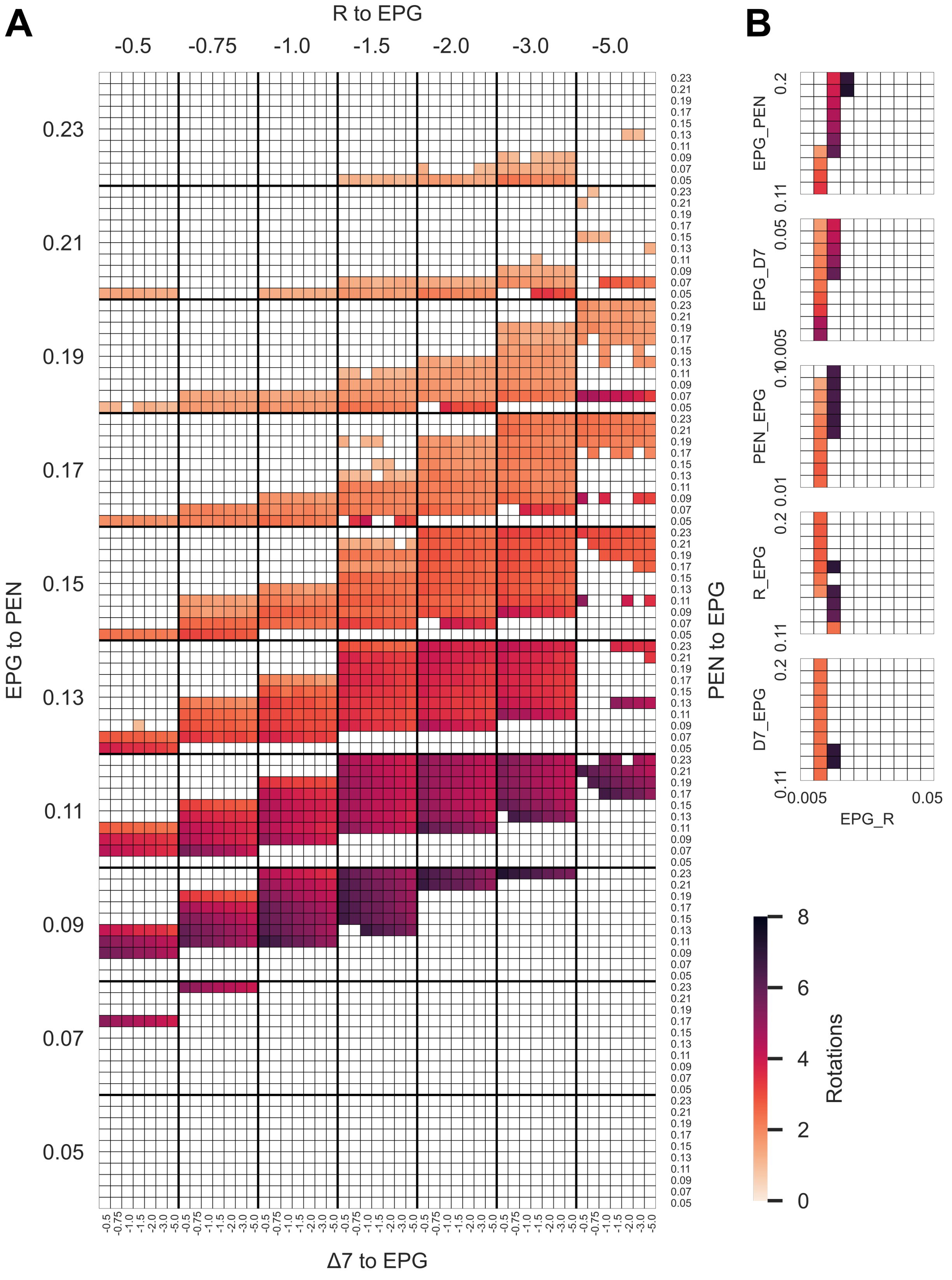


Fig S4: **Bump movement produced by different model parameters.** (A) In each row and column, the number of bump rotations for a single run of the model for a specific combination of weights is represented by colour (darker = more rotations; empty means weight combination was not viable). Bump rotations are measured by unwrapping the head direction estimate and finding the total degrees turned. On the x-axis, there are 7 sets of 7 columns. Each set of 7 columns has a fixed weight from R to EPG cell ranging from -0.5 to -5, shown on top x-axis. Within each set, the weight from Δ7 cells to

Fig S4: **(cont.)** EPG cells cycles through the same 7 values between -0.5 and -5, shown on the bottom x-axis. On the y axis, the weight from EPG to PEN cells varies every 10 sets of 10 rows (range 0.05 to 0.23 in steps of 0.02; shown on left y-axis) while the weight from PEN to EPG cells changes every cell (cycling in 10 steps from 0.05 to 0.23), shown on the right y-axis. Only parameters that meet the following conditions were considered viable: the bump must persist for the full simulation; the bump must be at least 2 cells wide and no wider than 8 cells; and the bump must move at least one full rotation within a 20000ms simulation. The parameter set in Table 1 results in 3 rotations in 20000ms. Selecting alternative parameters could allow different ranges of angular velocity to be accurately tracked. For all parameter combinations the weights from EPG to R, EPG to $\Delta 7$ and EPG to EPG are set to constant values specified in Table 1. (B) Number of rotations produced by the model when varying EPG to R weights (between 0.005 to 0.05 in steps of 0.005; x-axis). In each subpanel, the weight between two other groups of cells is varied (y-axis). The weights that were not varied were fixed to the values shown in Table 1.
