# Supplementary material for: Estimating orientation in natural scenes: A spiking neural network model of the insect central complex
Source: PLoS Comput Biol. 2024 Aug 15;20(8):e1011913. doi: 10.1371/journal.pcbi.1011913 (PMC11349202; doi:10.1371/journal.pcbi.1011913)
Supplement: Attachment — Submitted filename: 240627_responses.pdf [file pcbi.1011913.s005.pdf]

Reviewer #1: The paper presents a computational model focusing on orientation or heading direction within the insect central complex. While the core model shares similarities with several existing models in the literature, it incorporates more realistic visual inputs derived from recent experimental data (specifically, R2 and R4 neurons as used in this study). Tested with both simple stimuli (e.g. bright vertical bars or a linear array of triangles) and panoramic videos recorded from a moving camera in real environments, the model demonstrates its ability to estimate heading even with complex visual inputs, achieved through filtering by a small number of ring neurons with simple receptive fields, with the help of synaptic learning. While naturalistic visual inputs remain very limited, this represents a step forward. While the paper still exhibits some weaknesses, addressing them in a revision could further strengthen its contribution to the field.

Detailed comments:

The authors claim to introduce a novel spiking neural network model of the insect central complex. While the network itself may not be inherently novel given the anatomical constraints, the true innovation likely lies in a more realistic presentation of visual inputs. To better clarify the contribution of this paper, the authors should provide a more accurate description of the differences.

- As the reviewer points out, our largest contribution is in the more naturalistic representation of visual scenes by filtering with ring neuron receptive fields based on those recorded in drosophila, but also the types of complex natural visual inputs our model is capable of learning. We have modified the introduction to more clearly present how we are representing visual inputs to the model, and also emphasise the complexity of the natural visual inputs presented to the model. (L87-96,108-117)

Regarding synaptic weight learning, the results presented are based on only a few revolutions. However, considering real animals may experience numerous rotations over a reasonable timeframe, it would be valuable to explore the mature weight patterns after learning from many revolutions.

- One of the draws of taking inspiration from insects is how with small brains (~10^6 neurons) and low-resolution eyes (~10^3 pixels), insects learn to navigate in highly dynamic environments extremely rapidly and robustly. In this work we show in only one or two revolutions in a complex scene, it is possible to learn quickly an appropriate representation of visual information to support maintaining an accurate estimation of heading. In further revolutions the weight space becomes more stable and fewer weight changes occur. We have added two further panels to supplementary figure 3 to show how the weight space changes over revolutions. After 6 revolutions the weight space is fully developed and very few changes occur. (L224-226)

- As in our circling experiments the same visual scene is always encountered at each heading, learning is strengthened without conflict over many revolutions. No doubt future work with flies and in silico will explore the long-term dynamics of the circuit across large visual changes.

The authors should consider systematically exploring the parameter space, including testing the effects of weight strength on the results.

- We have performed a systematic search over the model parameters for weights between cell populations and included the outcome in the methods section. (L258-275)
- We can see that movement of the bump can be achieved for a range of parameter sets, with only one parameter (EPG cells to R cell weight) having a very narrow window of viability. (Figure S4)

Furthermore, changing the rotation angular speed or utilizing a naturalistic angular speed distribution may impact learning, particularly given the spiking-timing-dependent rule.

- As the reviewer suggested, the speed of bump movement does impact model performance somewhat. We have added a plot to show RMSE in the probe trial of the 3 rotation experiment at different angular velocities (Fig 3G). Current input to the model for each angular velocity was estimated using a curve fit to the data in Figure 3E which shows input currents required to generate different angular velocities of bump movement.
- ‘At high angular velocities less learning is able to take place before the probe trial, resulting in diminished performance. At low angular velocities learning occurs rapidly but bump movement is slow and there is more opportunity for developing unhelpful or overly strong mappings between ring neurons and EPG cells. This highlights the need for a variable learning rate at different angular velocities, evidence for which has been observed in Drosophila (Fisher 2022).’ (L329-343)

A closer examination of failed simulations for specific natural scenes where the activity bump was lost is warranted. Understanding the reasons behind these failures beyond correlational studies would provide valuable insights.

- Close inspection of the panoramas in the high failure group did not reveal any obvious patterns such as proximity to landmarks, sun visibility, camera shake, or density of forest, which could explain why these scenes performed worse than others. (L411-417)
- We asked neutral observers if there is anything that distinguishes the images from the successful and failure groups, and they also did not find any obvious differences.

The effect of translational movement of the camera on the learning process appears to depend crucially on the distance from the camera to the landmarks. Therefore, documenting the distribution of distances from the camera to nearby objects is essential. Even crude estimates would prove useful for other researchers.

- We will consider this comment carefully for future work, where the camera is moved over longer distances, and keep track of distances to major landmarks. However, because ring neuron activation depends on bright areas of the visual scene, it is actually gaps between landmarks like trees that result in high activation. Landmark position may become more important if we include other ring neuron types such as ER4d cells which represent 'motion-defined cues' as described by Duan et al. (2023).
- As in the circling case each heading is only experienced at one position of the trajectory, it is unlikely that the change in position of landmarks relative to the camera will have an impact on performance because the view will always be the same when a heading is revisited. However the movement of the camera does cause increased camera shake and in some videos large distortions during some frames which may better explain increased error for these videos.
- As the reviewer suggested we have added qualitative statements to Figure S2 about the distance to the closest landmark

Fig. 2A:
some labels at the top (90 deg rotated) need more spacing
Need consistent notation: label D7 in Fig. 2 Vs delta 7 in Fig. 1

- We have revised Figure 2 to improve legibility and consistency of delta7 labelling.

Fig 5:
B: Why are there pixel correlations at 180 deg? Bias in the video? It may cause confound with the ring activity correlation.
The organization of the panel labels is confusing. For example, "G: High correlations between ring neuron activations…" seems wrong.

- Manuscript revised for clarity. As the reviewer suggests, there are regions where correlation is not zero at intermediate headings, which is what we were trying to point out in panel G especially. This comes about from ambiguity and aliasing in the natural scene visual input.
- The non-zero correlations around 180 degrees may correspond to visual features such as gaps in the tree cover at either end of a path. However it seems these non-zero correlations in the pixel value or in the ring neuron activations do not result in the model failing to track heading. (L372-380)
- We have also revised the figure legend to essay 'Non-zero' correlations rather than high correlations at other headings.

Reviewer #2: The authors propose a ring-neuron network model with synthetic receptive fields with STDP (spike-timing dependent plasticity) for the insect central complex. They demonstrate that their model is able to reproduce observed neural activity and behavior under simple and natural scenes. Previously, neural network models of this brain area mostly focused on the function of the attractor dynamics and how a bump can be formed under simple stimuli. Other models showed how plastic ring circuits can form a map between visual stimuli and the ellipsoid body. This work fills the gap and provides a more complete picture of the mechanism of visual orientation of fruit flies. However, there are several critical issues the authors need to address. Here are my specific comments:

Major comments

1. The hypothesis of the hexagonal arrangement of the receptive fields of the R2 and R4 neurons seems crucial for the model. Is there any experimental evidence for the hypothesis? Otherwise, the authors should also test other arrangements, such as square lattice or random spacing. If the hexagonal arrangement delivers the best performance, this can be a testable prediction of the model.

- As we were including more ring neurons in the model than had known receptive fields recorded in Drosophila, we needed a method of positioning the fields in the visual scene. We selected a hexagonal arrangement of receptive fields as this is an efficient way of packing circular receptive fields, however this arrangement is not essential to the model. As the reviewer suggested we show in Fig 3G RMSE in the probe trial of the 3 rotation experiment with the bar stimulus for hexagonal, square grid and random RF placement for different angular velocities. (L177-182)
- We have also included examples of the simulated RFs for each of these arrangements in Fig S1, in all cases RFs that are not dissimilar to those recorded in drosophila are produced.

2. The authors referred to Vogels et al 2011 for their model of plasticity for inhibitory synapses. However, unless I understand it incorrectly, the plasticity model in Vogels et al 2011 (Eqs 3-5 in Supporting Material) exhibits an opposite effect to what the authors modeled in the manuscript. Vogels et al 2011 used conductance-based synapses. Their weights represent synaptic conductance and are always positive. When postsynaptic and presynatpic spikes occur within a small time window, the weight increases, leading to strengthened inhibitory synapses. However, in this manuscript, the authors appeared to use current-based synapses, so the inhibitory weights are negative. When coincident spikes occur, the weights become less negative, or the inhibitory synapses are weakened. Can the authors elaborate? Is there any experimental support for this uncommon STDP rule?

- We have implemented the type of anti-Hebbian learning expected at these synapses described previously in numerous publications (eg. Fisher et al. 2019, Okubo et al. 2020, Fisher 2022, Kim et al. 2019). The reviewer is correct that the learning in Vogels et al. (2011) is a Hebbian rule where coincidently timed spikes lead to stronger inhibition at those synapses, rather than the reduced inhibition seen in our model.
- We have implemented the learning rule in such a way that coincident spikes result in the weight movement towards zero - a more positive (larger) value than the original weight (adding a positive number to the weight), and the order of the spikes (pre- then post-synaptic or post- then presynaptic) does not matter. In this way we are implementing a very similar rule to Vogels et al. (2011), see figure Figure 1C.
- We have clarified this in the manuscript by adjusting the sentence to say ‘inspired by the symmetric shape presented in Vogels et al. (2011). (L217)

Fisher YE, Lu J, D’Alessandro I, Wilson RI. 2019 Sensorimotor experience remaps visual input to a heading-direction network. Nature 576, 121–125. (doi:10.1038/s41586-019-1772-4)

Okubo TS, Patella P, D’Alessandro I, Wilson RI. 2020 A neural network for wind-guided compass navigation. Neuron 107, 924–940. (doi:10.1016/j. neuron.2020.06.022)

Fisher YE. 2022 Flexible navigational computations in the Drosophila central complex. Curr. Opin Neurobiol. 73, 102514. (doi:10.1016/j.conb.2021. 12.001)

Kim SS, Hermundstad AM, Romani S, Abbott LF, Jayaraman V. 2019 Generation of stable heading representations in diverse visual scenes. Nature 576, 126–131. (doi:10.1038/s41586-019- 1767-1)

3. It appears that the model has the best performance when the bump is solely driven by the visual input from the ring neurons and the error increases when the model takes additional input from PEN neurons (see Fig 3A, 4B, 5D & 5J). Why is that? Did the authors test whether they had a correct PEN input strength? Because the moving speed of the bump is also modulated by the PEN input, if the input strength is too high or too low, it might interfere with the ring-neuron-driven bump movement.

- It may be the case as the reviewer pointed out that a better PEN input strength could be found. However in the case that there is no ring neuron input, current input to the model produces low error over 3 revolutions, more closely matching the ground truth in the first

two rotations than when the ring neuron input is available.

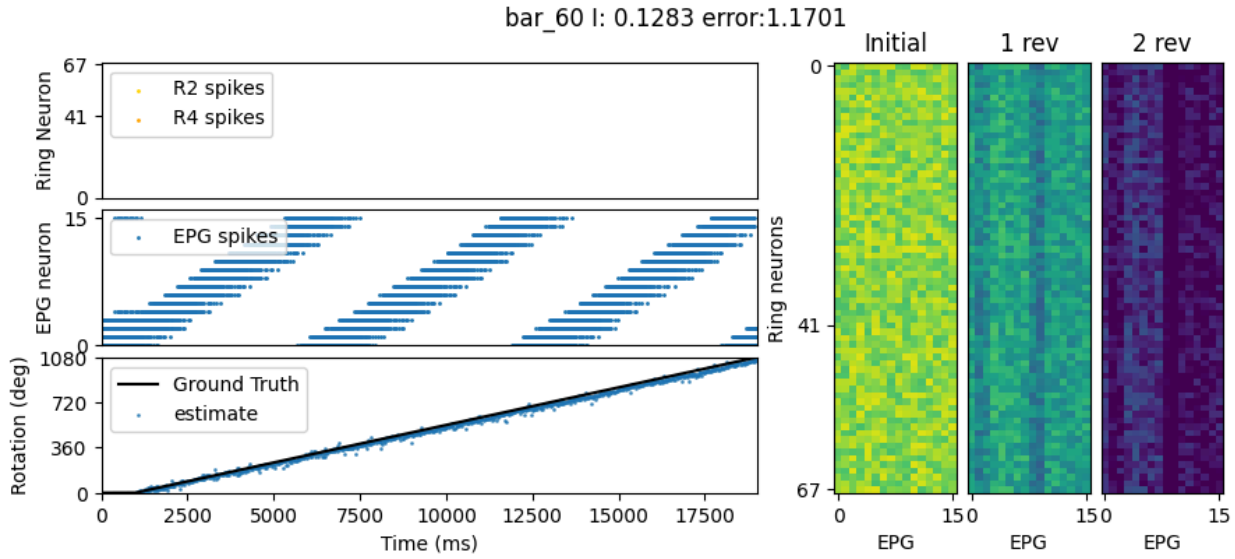

- With additional input from ring neurons, the bump position is influenced from the beginning of the simulation by ring neuron input due to the random initial weights. The random initial weight space also leads to variability in the outcome of the simulation seen in Fig 5.
- We have added a plot in figure 3F that shows the RMSE across 3 revolutions when only PEN input is provided, and the curve used to estimate appropriate current input to achieve different angular velocities (Fig 3E)

4. What happens if the virtual scene has a bright (white) background with one or two black stripes? This type of visual stimuli has been used widely in fruit fly behavioral tests. Flies are shown to navigate and remember the location of the stripes without problem. Does the model proposed by the authors perform equally well or is it unable to keep the bump due to the strong activation of most ring neurons? I would guess the latter, but I could be wrong. This can be a good way to validate the model.

- When applying the model to a scene with a black bar on a white background the bump is lost due to strong activation of ring neurons as the reviewer predicted.
- Even drastically increasing the inhibitory weight strength between ring neurons to encourage fewer active cells does not improve performance. We suggest in this scenario further image processing by upstream regions of the visual system must be involved to prevent saturation of ring neuron activity.
- Alternatively this type of visual input may be better represented as a ‘Motion-defined cue’ as described by Duan et al. (2023) which is represented by a further population of ring neurons (ER4d). We will consider this carefully for future work, and may add additional ring neuron types or more detailed visual processing by upstream regions.
- The white bar stimulus on a black background is more similar activations-wise to the forest scenes we were interested in.

5. It is a cool idea to test the model using triangles with different arrangements and the result is very impressive. However, not being able to discriminate between the objects (as tested in Ernst & Heisenberg 1999) is not equivalent to not being able to keep orientation (as shown in this study). It is likely that they are two completely different neural mechanisms responsible by different neural pathways. The authors need to acknowledge and discuss this in the Discussion section, or simply remove this part.

- This triangle discrimination experiment was intended to show another example of similarities between our work and previous experimental work, increasing confidence in the model. However, we acknowledge that by providing the triangle stimuli as visual input to the model we are going beyond the results of the original work. We show that because of the lack of discriminability by ring neurons, replicating what was observed experimentally, this type of stimulus is also not suitable for forming a useful mapping between ring neurons and EPG cells, such that this stimulus can be used to track heading.
- However, as exploring this further is not the intended direction of this manuscript and including this result opens up further questions (see next comment from reviewer), we have removed this result and associated discussion as suggested by the reviewer.

6. Following the comment above, I guess the inability to move the bump (Figure 4C) may be just a coincidence stemming from the specific sizes and arrangements of the cues and the hexagonal receptive fields. What happens if the size of the triangles and the space between them are changed? My main point is that if the authors want to keep this part, they should investigate the mechanism underlying such indiscriminability rather than just showing the phenomenon.

- As suggested by the reviewer, we have removed the triangle discrimination experiment as it was not achieving its intended purpose of increasing confidence in the model but instead opened up further questions in an unintended direction.
- However we do know that the indiscriminability was not due to the hexagonal arrangement, because in a very different piece of work Dewar et al (2015) showed the the Ring neuron code was ambiguous for those triangle stimuli

Dewar ADM, Wystrach A, Philippides A, Graham P. Neural coding in the visual system of Drosophila melanogaster: How do small neural populations support visually guided behaviours? PLOS Computational Biology. 2017;13:e1005735. doi:10.1371/JOURNAL.PCBI.1005735.

Minor comments

1. Line 113. Please specify the unit of tau. If the unit is millisecond, 50 ms for inhibitory

synapses is way larger than typical GABAa receptors, unless the authors modeled GABAb. 100 ms for the excitatory synapses are also larger than what would be expected for cholinergic receptors. Is there any support for such large synaptic time constants? Is there any specific reason why excitatory synapses need longer time constants than that of inhibitory synapses? I can understand that longer time constants are crucial for stabilizing the dynamics of a neural circuit with a small number of neurons. The authors need to explain the reason in Methods or discuss it in Discussion.

- Thank you for highlighting this. The unit of tau is indeed ms (updated in the manuscript). The time constants we use in this work are within the range of GABAb and potentially NMDA, however, with full transparency, we selected these values to stabilise the circuit dynamics as the reviewer suggested. We are able to find parameters for shorter time constants 5ms inhibitory and 20ms excitatory which may more accurately reflect GABAa and cholinergic receptors respectively. However the parameter space is much more fragile and the model much less stable. (L129)

2. Figure 2 legend, third line from bottom: missing the figure number in "(see Figure)"

- Corrected in manuscript

3. Figure 2B. Please label the neuron indices (all or a few representative ones). Otherwise, without known which neuron is which, the connection matrices in panel C are meaningless.

- We have labelled the RF at the end of each row to make identification clearer and expanded the explanation in the figure legend.

4. Line 263: The phrase "median active EPG cell" is confusing. Using the word "median" makes people think that the author sorted the EPG cells by their activity level and choose the middle one.

The centre of the activity bump was found by:

1) Bin spike times
2) Unwrap the bump
3) For each time bin find the active cells
4) Then find the median active cell - the middle cell of the bump

For clarity we have replaced mentions of the median active cell with 'selecting the EPG cell at the centre of the active cells' in each bin. (L305)

5. Lines 323-333. Some sentences are too long and poorly written. Please rewrite them.
6. Line 332. "…resulting in multiple peaks." Did the author mean several activity bumps in EPG cells? I do not see them in the figure.

- We meant to refer to multiple peaks in the correlation matrix but this was not clear. Manuscript revised for clarity. (L370-380)

7. Line 352. “Is” it.
8. Line 358. …in the error “measured” across all simulations….
9. Line 359. “… greater than 1% failed simulations.” Do the authors mean that they run more than 100 trials and count the number of failed trials? What do they define a "failed simulation?"

- Simulations were considered a failure if the bump collapsed and the centre could not be measured. If more than 3/320 simulations performed for each scene failed these scenes were added to the high failures group. Manuscript revised for clarity. (L411-413)

10. Figure 6. The authors may want to display a few example scenes for high, low variences and failed simulations.

- Manuscript revised to add a label to each of the panoramas shown in figure S2 showing the group each falls into.

11. The authors use the phrase “multi-polar ring neurons” several times in the text. However, it is confusing because “uni-polar”, “bi-polar” and “multi-polar” are usually used to describe the morphology of a neuron. The authors may want to find a better phrase for what they want to describe.

- Replaced with multi-directional ring neurons for clarity.

12. Line 378. “However” --> should start a new sentence.
13. Line 378. “…all groups…” What groups? High, low variances and failed simulations?
14. Line 277-382. Again, a very long sentence hard to comprehend. Please rewrite it.

- For clarity we have revised the results section to use shorter sentences.
- We particularly focused on improving clarity between lines 311-327

Have the authors made all data and (if applicable) computational code underlying the findings in their manuscript fully available?
The PLOS Data policy requires authors to make all data and code underlying the findings described in their manuscript fully available without restriction, with rare exception (please refer to the Data Availability Statement in the manuscript PDF file). The data and code should be provided as part of the manuscript or its supporting information, or deposited to a public repository. For example, in addition to summary statistics, the data points behind means, medians and variance measures should be available. If there are restrictions on publicly sharing

data or code —e.g. participant privacy or use of data from a third party—those must be specified.

Reviewer #1: Yes

Reviewer #2: **No:** Unless I miss it, I do not see the authors mentioning any link to their code in the manuscript.

- A link to the code was provided during the manuscript upload, we are not certain if the version provided to reviewers included this statement.
- Code can be accessed here: https://github.com/stenti/stentiford_cx_ra
